# Soil Microbial Dynamics in Regenerative Agriculture Systems: A Data-Driven Synthesis for Soil Health, Pest Suppression, and Yield Sustainability in the Western Canadian Prairies

**DOI:** 10.3390/microorganisms14051075

**Published:** 2026-05-09

**Authors:** Susmita Das Nishu, M. Nazrul Islam

**Affiliations:** 1 Department of Environment and Sustainability, University of Saskatchewan, Saskatoon, SK S7N 5A2, Canada; susmita.nishu@usask.ca; 2 College of Graduate and Postdoctoral Studies, University of Saskatchewan, Saskatoon, SK S7N 5A2, Canada

**Keywords:** regenerative agriculture, reduced tillage, diversified rotation, cover cropping, soil microbial dynamics, western Canadian prairies, soil health, pest suppression, yield sustainability

## Abstract

Regenerative agriculture (RA) is expanding across the Western Canadian Prairies, but its microbial foundations under climatic constraint remain insufficiently integrated. This review synthesizes evidence from long-term Prairie field experiments, regional and global datasets to evaluate how regenerative management reshapes soil biological processes and agronomic performance across systems. RA practices including no-till, diversified rotations, cover cropping, and organic amendments consistently enhance microbial biomass (up to 40–86%), arbuscular mycorrhizal fungal abundance (32–60%), and microbial diversity (≈50%), alongside increases in soil organic carbon (up to 15.6 kg C ha^−1^ yr^−1^), aggregate stability (up to 38%), and water retention (up to 30–34%). These biologically mediated improvements are linked to enhanced nutrient cycling and crop nitrogen uptake (13–47%), as well as increased microbial enzymatic activity and functional gene abundance. Agronomically, these changes translate into yield gains ranging from 10% to 147% under long-term no-till and 14–38% under diversified rotations, with additional system-level benefits including reductions in synthetic nitrogen inputs (up to 73%) and herbicide use (up to 42%). While agronomic benefits vary across temporal scales and environmental conditions, this synthesis identifies microbial communities as key mediators of interactions among climate, plant, and soil systems, underpinning improvements in soil health, pest suppression, and yield stability in semi-arid, climate-variable Prairie agroecosystems. Continued long-term, system-level research is needed to refine regionally adapted regenerative transitions and to clarify how microbial processes mediate resilience under future climate uncertainty.

## 1. Introduction

Soil microorganisms are key drivers of the core processes that sustain agroecosystems, including nutrient cycling (e.g., nitrogen fixation, phosphorus solubilization), organic matter decomposition, soil water retention, toxic compound degradation, and the suppression of plant pests and pathogens [1]. These interlinked ecological functions form the biological foundation of Regenerative Agriculture (RA), an outcome-driven, systems-based approach that integrates natural processes into farming to enhance soil health and promote resilient, sustainable production systems [2]. Despite their critical role as key biological mediators linking management practices to soil functions and agronomic outcomes, soil microbial communities remain underemphasized and insufficiently characterized within Prairie agroecosystems.

This gap in understanding is particularly consequential in the Canadian Prairie region, one of the world’s largest contiguous agricultural landscapes and a major contributor to global food security. The widespread adoption of high-yielding crop varieties, extensive monocultures, synthetic inputs, and mechanized farming has dramatically increased food production but has also left a legacy of ecological disruption in Prairie agroecosystems [3]. Soil deterioration, organic matter and biodiversity loss, poor water management, and greenhouse gas emissions now threaten long-term sustainability and increase vulnerability to pest and disease outbreaks [4]. These pressures are intensifying under climate stress, as prolonged cold winters, relatively short growing seasons, recurring droughts, erratic precipitation, and temperature extremes increasingly constrain yield stability across semi-arid to sub-humid Prairie zones [5]. Within this context, understanding how microbial communities buffer or amplify these stresses becomes critical for designing effective RA strategies.

These climate and management pressures are further shaped by inherent soil properties across the Prairie region. Prairie soils are dominated by Chernozemic, Luvisolic, and Solonetzic orders, which are particularly sensitive to management because their structure, nutrient retention, and water-holding capacity depend strongly on organic matter and microbial activity. Agriculture and Agri-Food Canada’s Soil Organic Carbon Change indicator reported that between 1981 and 2016, approximately 60% of prairie cropland experienced measurable SOC decline [6]. This pattern is consistent with site-level analyses reporting SOC loss, particularly where perennial grasslands were converted to annual cropping or wetlands were drained [7,8]. By contrast, lands with early adoption of no-till and diversified crop rotations showed SOC stabilization or modest recovery, indicating that degradation is management-driven rather than inevitable. These contrasting trajectories indicate that microbial processes governing SOC and aggregation are highly responsive to how prairie soils are managed.

In response to these degradation patterns and their underlying microbial drivers, sustainable practices such as cover cropping, reduced tillage, and holistic grazing management in the Canadian Prairies have been reported to rebuild soil organic matter, enhance carbon sequestration, and reduce greenhouse gas emissions [4]. Adoption of RA practices has therefore emerged in Western Canada not as a novelty but as a necessary corrective response, supported by government initiatives such as the Sustainable Canadian Agricultural Partnership [9]. Although Canada is not yet the global leader in conservation tillage, approximately 65% of arable prairie land is under no-till systems [10]. Cover cropping has also gained momentum, with 2020 surveys reporting that 281 farmers across the Canadian Prairies cultivated over 41,000 ha of cover crops, despite regional climatic challenges [11]. Taken together, these trends underscore that farmers are already shifting disturbance, residue, and grazing regimes in ways that implicitly rely on microbial recovery, even if microbial responses are not explicitly measured.

Despite growing adoption, key gaps remain in understanding microbial functioning in these systems. With rising climate risk, input costs, and ongoing soil degradation, Prairie agriculture faces an urgent need for biologically grounded solutions that deliver both resilience and productivity. However, most syntheses remain narrowly focused on individual practices such as no-till, without integrating how multiple RA practices jointly restructure microbial communities and the processes they control. As a result, a comprehensive, data-driven understanding of how soil microbiomes mediate soil health, pest suppression, and yield stability under RA remains limited. This review addresses that gap by synthesizing long-term field studies and prairie-scale experiments to clarify how RA practices regulate microbial processes that stabilize yields and rebuild soil function. Specifically, we aim to: (i) synthesize data-driven evidence linking RA practices to microbially mediated improvements in key soil health indicators (e.g., SOC, aggregation, nutrient cycling); (ii) elucidate microbial mechanisms underpinning enhanced crop yield and stress resilience in Canadian Prairie systems; (iii) evaluate how microbial regulation under RA contributes to weed, insect, and pathogen suppression; and (iv) identify research gaps and future directions for microbiome-informed RA tailored to Western Canada.

To address these objectives within a heterogeneous evidence base, this study adopts a data-driven, cross-study synthesis to evaluate soil microbial dynamics and agronomic outcomes under regenerative agriculture (RA) in the Western Canadian Prairies. Given the substantial heterogeneity in experimental design, soil properties, climatic conditions, and measurement approaches across studies, we employed a structured case-by-case synthesis rather than a formal meta-analysis. We assess the effects of RA practices on physical, chemical, and biological soil indicators across short- and long-term temporal scales in prairie agroecosystems. Measurable soil biota attributes, including microbial colonization, biomass, diversity and composition, soil respiration, enzyme activities, and functional genes, are referred to as biological indicators. Statistical results were interpreted as reported in the original studies, without applying cross-study normalization or additional assumptions. Reported percentage changes (e.g., microbial biomass, yield, pest suppression) were therefore used as relative indicators to compare directional responses across systems. Within this framework, the synthesis emphasizes identifying consistent patterns, explaining sources of variability, and highlighting functional trade-offs, rather than deriving uniform estimates. This approach allows for a more context-sensitive interpretation of microbial responses across environmental and management gradients while preserving the integrity of individual study designs. We propose that RA enhances yield stability and resilience in Prairie agroecosystems by reorganizing soil microbial communities, which interact with plant and climatic factors to regulate ecosystem functioning. Particular emphasis is placed on explaining sources of variability, identifying conflicting findings, and clarifying context dependency (e.g., soil type, climate, and temporal scale) across studies.

## 2. A Century of Pressure on the Prairie Breadbasket

**Figure 1:**
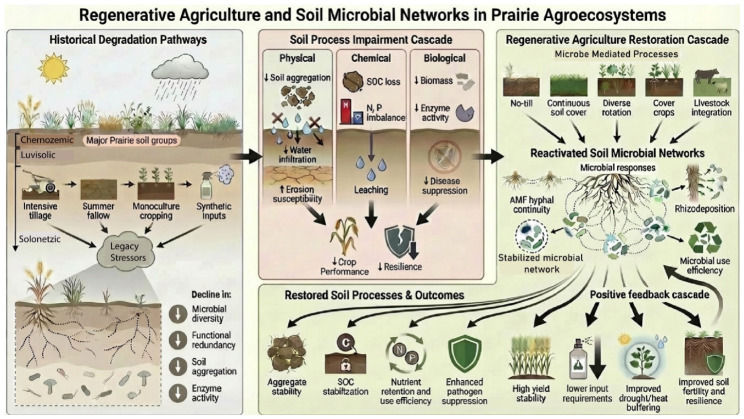
Conceptual framework for regenerative agriculture (RA) in Canadian Prairie agroecosystems. RA practices (reduced/no tillage, diverse rotations, cover crops, organic amendments, crop-livestock integration) counteract historical degradation by improving microbial habitat and networks, which in turn enhance soil structure, SOC stabilization, nutrient and water dynamics, pest suppression, and long-term yield stability while gradually lowering input needs.

The Canadian Prairie provinces, spanning Alberta (AB), Saskatchewan (SK), and Manitoba (MB), approximately encompass a vast region of 52.6 M ha, comprising 81% of Canada’s agricultural land [12]. Producing over 80% of Canada’s field crops, the Prairies are widely recognized as a global “breadbasket” [13]. However, as illustrated in Figure 1, more than a century of intensive tillage, summer fallow, monoculture cropping, and heavy agrochemical use, compounded by growing climate stress, has progressively weakened soil structure, depleted soil organic carbon (SOC), and eroded agroecosystem resilience. Within this setting, the historical degradation pathways in Figure 1 frame the central question of this review: whether rebuilding soil microbial networks can shift Prairie agroecosystems from vulnerability toward recovery.

### 2.1. Climate Constraints and Agronomic Vulnerability

Climatic constraints in the Canadian prairies place continual stress on soils and crops, making the region highly dependent on the buffering capacity of soil biota. The Canadian Prairie region experiences a continental climate with long winters, short growing seasons, and pronounced interannual variability in precipitation and temperature [14]. Recurrent droughts, heat waves, and episodic heavy rainfall increasingly affect crop productivity and exacerbate soil degradation processes, such as erosion and moisture loss [13]. Between 2009 and 2019, severe weather events across Canada resulted in an average of $1.9 billion in insured damage claims annually [13]. In Prairie systems, yield stability depends more on soil biological buffering capacity than on short-term inputs, a central message of Figure 1. RA practices minimize soil disturbance, improve infiltration and nutrient retention, and consistently enhance microbially mediated processes, which in turn mitigate climate risk by buffering soil moisture and temperature extremes rather than simply increasing mean yields [11,12,13]. Thus, climate risk makes the microbial “buffering layer” depicted in Figure 1 not a short-term input but a critical target for regenerative management.

### 2.2. Soil Organic Carbon (SOC) Depletion and Legacy Effects

Historically, prairie soils supported high SOC stocks and diverse microbial communities due to native grasslands and deep perennial rooting systems. The twentieth-century agricultural intensification yield-maximization paradigm disrupted this equilibrium [15]. As depicted in Figure 1, repeated tillage, summer fallow, and land conversion caused widespread SOC loss, compaction, and salinity expansion, especially in fine-textured and marginal soils [15,16]. In Saskatchewan, the area under no-till expanded from just 8% in 1981 to 73% by 2016, contributing to a regional increase in SOC index values from 48 in 1981 to 78 in 2006 before stabilizing near 72 in 2016 [6,17]. Continuous wheat and summer fallow rotations in Swift Current, SK, lost up to 20–25% of SOC over three decades, compared with diversified rotations and perennial phases that maintained or slightly increased SOC [18,19]. These outcomes reinforce that SOC decline is a legacy effect of disturbance rather than an inevitability and imply that RA strategies that rebuild microbial activity and carbon inputs can partially reverse this trajectory.

### 2.3. Monoculture and Biological Simplification

Economically successful but biologically simplified systems now dominate prairie agriculture, with cereals (e.g., wheat, barley), oilseeds (canola), and pulses (lentils, peas) occupying most cropped land [13]. While economically effective, monocultures and intensive tillage have led to biological homogenization and soil nutrient depletion [15]. Field studies show that cereal- or canola-dominated systems reduce microbial richness and favor generalist taxa adapted to low-diversity environments, while suppressing beneficial guilds such as arbuscular mycorrhizal fungi and antagonistic bacteria [15,20]. Reduced microbial niche availability limits functional redundancy, increasing vulnerability to disease outbreaks and climate stress. Thus, monoculture represents a microbial bottleneck that undermines long-term soil function and resilience in Prairie agroecosystems, and it motivates the RA focus on diversified rotations and continuous cover in Figure 1 as key levers for microbiome repair.

### 2.4. Reliance on Synthetic Input

A parallel trend has been the heavy reliance on synthetic nitrogen, phosphorus, and pesticides, which has further reshaped Prairie soil ecosystems and their microbial communities. Excess nutrients favor fast-growing copiotrophic microbes while suppressing oligotrophic taxa that contribute to long-term carbon stabilization and nutrient-use efficiency [21,22]. Similarly, fungicides and herbicides can exert off-target effects on non-pathogenic fungi and bacteria, disrupting symbioses and reducing functional diversity [22,23]. In semi-arid Prairie soils, where SOC is already limited, this chemical dependence amplifies soil degradation, increasing reliance on external inputs and weakening microbial resilience. These patterns emphasize the need for management approaches such as RA that restore biological, especially microbial, regulation of nutrients and pests rather than attempting to replace it indefinitely with chemical substitutions.

### 2.5. Impacts on the Hidden Life Beneath Prairie Soils

Within this degraded and chemically dependent context, the “hidden” microbial life beneath Prairie soils emerges as a sensitive integrator of management and climate. Soil microbial activity is closely linked to soil moisture, temperature, and organic inputs. Drought years consistently reduce microbial biomass and respiration, particularly among decomposers and nitrogen-cycling taxa [24]. Prolonged stress shifts communities toward stress-tolerant taxa, often reducing functional redundancy and ecosystem stability [25]. Lupwayi et al. (2001) found that conventional tillage caused greater microbial biomass losses in acidic, carbon-poor Luvisolic soils than in carbon-rich Chernozemic soils, highlighting the buffering role of baseline SOC [26]. Across Prairie systems, tillage intensity often exerts a stronger influence on microbial community structure than fertilizer inputs, favoring opportunistic bacteria and pathogens over symbiotic fungi such as AMF [27]. Crops also shape microbial communities through root exudates, residue inputs, and rotation patterns. Monocultures narrow community composition, whereas diversified rotations expand niche availability and support specialized guilds involved in nutrient cycling and disease suppression [28]. These observations align with Figure 1, positioning soil microbes as key mediators linking management decisions to long-term soil function and resilience, and providing a mechanistic bridge to the next section, where regenerative practices are treated as deliberate attempts to re-engineer these microbial networks in prairie systems.

Microbial functional groups mediate the mechanisms by which regenerative agriculture (RA) enhances soil function and crop performance. Bacterial communities respond rapidly to RA practices, accelerating labile carbon decomposition and nitrogen transformations (e.g., mineralization, nitrification), thereby improving short-term nutrient availability and synchrony with crop demand, particularly under pulse disturbances [1,29]. In contrast, arbuscular mycorrhizal fungi (AMF) are favored under low disturbance and continuous plant cover, enhancing phosphorus acquisition and water uptake through hyphal networks while contributing to aggregation and soil organic carbon stabilization via hyphal enmeshment and glomalin production [27]. Bacterial dominance prevails in high-input systems, whereas AMF-mediated functions become critical under low-disturbance, moisture-limited conditions typical of Prairie environments. Together, these fast (bacterial) and slow (fungal) pathways position microbial communities as key regulators of climate–plant–soil interactions and overall system resilience.

In the Western Canadian Prairies, where agroecosystems are frequently exposed to drought and episodic excessive rainfall, soil microbial dynamics are strongly regulated by soil moisture variability. Under drought, reduced soil water limits substrate diffusion and microbial activity, leading to declines in microbial biomass and nutrient mineralization, while selecting for drought stress-tolerant taxa [30,31]. In contrast, excessive rainfall, particularly in fine-textured or poorly drained Prairie soils, can induce transient waterlogging and oxygen limitation, favoring anaerobic taxa (e.g., denitrifiers, methanogens), shifting microbial communities from aerobic to anaerobic metabolisms, altering community composition and biogeochemical processes [32].

### 2.6. Framework of Prairie Regenerative Agriculture

In response to the long-term pressures outlined above, Prairie farmers and researchers have been applying principles now labelled “Regenerative Agriculture” since at least the 1970s, particularly through conservation tillage, residue retention, and crop diversification [33]. Guided by these six core principles (summarized in Supplementary Table S1), RA operates as a “stimulation–response–outcome” system; management practices stimulate microbial mechanisms, which drive ecosystem processes that ultimately shape agronomic and environmental outcomes.

Practices such as cover cropping, crop rotation, residue retention, and minimizing tillage effectively improve soil water-holding capacity, mitigate temperature extremes at the root zone, increase SOC and soil aggregation, thereby strengthening climate resilience in Prairie systems [34]. Empirical studies have shown that regenerative fields exhibit greater microbial activity, higher water infiltration rates, and reduced nutrient leaching compared to conventionally managed lands [35]. In the Prairie context, crop diversification and crop rotation sequence significantly contribute to both soil fertility and disease regulation. Pulse-based rotations enrich nitrogen-fixing bacteria (Rhizobium, Bradyrhizobium), increase microbial biomass and diversity, and suppress certain soilborne pathogens, contributing to both fertility and disease regulation [36]. Deep-rooted and diverse crops further extend microbial habitat into subsoil layers, improving structure and resource access [37]. Collectively, these findings demonstrate that RA in the Canadian Prairies is a biologically grounded strategy for restoring soil function, buffering climate risk, and sustaining long-term productivity, with microbial networks, highlighted in Figure 1, acting as a key pathway through which management changes translate into resilient agroecosystem outcomes.

## 3. Result and Synthesis

### 3.1. Effects of No-Till (NT) and Reduced Tillage (RT) on Soil

Table 1 provides a synthesis of long-term Prairie and temperate studies assessing how no-till (NT) and reduced tillage (RT) influence soil physical, chemical, and biological indicators relevant to RA. Reduced or minimum tillage (RT/MT) and no-tillage (NT) reduce soil disturbance, which restructures microbial habitats in prairie soil. Residue cover generates a buffered microenvironment by increasing soil moisture retention (+3.5–5.6% volumetric water content) and balancing temperature extremes, which support microbial survival, activity, and spatial continuity under the soil surface [38,39,40]. NT induces soil aggregation by fungal hyphae and microbial exopolysaccharides, increases macroaggregate formation (7–38%), and reduces wind-erodible fine aggregates [41,42]. Initial increases in surface bulk density and penetration resistance are generally shallow and decline over time as organic matter accumulates and pore connectivity improves [43,44]. Soil fertility responses reflect improved carbon and nutrient dynamics. Long-term NT increases soil organic carbon (2.14 g kg^−1^ in surface soils; 4 Mg C ha^−1^ over decades) through stabilization of recalcitrant pools [45]. Residue retention and crop diversification under NT enhance nitrogen uptake (13–47%), indicating stronger mineralization and retention within the soil–plant system [41,46]. Long-term NT alleviates pH stratification, reflecting a shift toward microbe-mediated nutrient distribution rather than reliance on mechanical mixing [47]. Biological indicators provide direct evidence of NT acting as a catalyst for microbial functional enhancement. The 25-year-long NT practice in the Prairie increases microbial biomass carbon up to 86% in the surface layer (0–5 cm), along with higher activities of enzymes involved in C, N, and P cycling (e.g., β-glucosidase, cellulase, xylanase, phosphatase) [48,49,50,51]. NT supports 32–60% greater arbuscular mycorrhizal fungal (AMF) biomass, maintaining hyphal networks that enhance nutrient acquisition and aggregation [10,49]. Functional gene abundance (e.g., *nifH*, *nirK*) further indicates stronger nitrogen cycling potential, although shifts in (*nirK* + *nirS*)/*nosZ* ratios suggest context-dependent trade-offs in N_2_O emissions under certain moisture conditions [52].

Table 1 reveals that the most consistent outcome of NT/RT is a gradual reorganization of the soil habitat rather than an immediate agronomic response. Reduced disturbance and residue retention enhance moisture stability, pore continuity, and carbon inputs, favoring fungal dominance, microbial biomass accumulation, and aggregation. However, these responses are strongly stratified by depth and time. Improvements in microbial biomass, enzyme activity, and aggregation are concentrated in surface layers where residues and roots are most active. In contrast, deeper layers often show weaker or delayed responses. Similarly, short-term studies frequently report increased surface bulk density or penetration resistance, reflecting the legacy of mechanical disturbance and the absence of rapid structural reformation. These constraints diminish over time as microbial processes restore structure, indicating that physical recovery lags behind biological restructuring.

The second key pattern is that NT/RT outcomes are highly context-dependent rather than universally beneficial. Variability across studies is best explained by interacting controls, including soil type, moisture regime, and duration of adoption. Carbon-rich Prairie soils and long-term systems tend to exhibit stronger benefits in soil organic carbon, microbial biomass, and AMF abundance, reflecting sufficient substrate availability and time for stabilization processes. In contrast, wetter or poorly aerated, carbon- and nitrogen-rich conditions can shift nitrogen cycling toward denitrification pathways, where increases in *nifH* and *nirK* do not necessarily translate into net ecosystem benefits. Instead, shifts in (*nirK* + *nirS*)/*nosZ* ratios indicate potential imbalances between N_2_O production and reduction, highlighting a trade-off under high moisture conditions [55]. However, gene abundance-based indices often show weak or inconsistent relationships with actual N_2_O fluxes, as process rates are strongly regulated by environmental conditions and microbial activity rather than genetic potential alone [56]. In semi-arid Prairie systems, where moisture is often limited, such conditions occur intermittently, resulting in episodic rather than sustained emissions, often following wetting events.

Collectively, Table 1 indicates that NT/RT drives a time-dependent reorganization of soil microbial habitats, with outcomes governed by site-specific constraints rather than a uniform trajectory of improvement. Divergent outcomes under NT/RT largely reflect edaphic controls (texture, mineralogy) and hydroclimatic variability that modulate carbon protection mechanisms and oxygen diffusion, thereby shifting microbial pathways between stabilization and gaseous loss. Apparent inconsistencies such as concurrent gains in microbial biomass and elevated N_2_O emissions or transient compaction effects are characteristic of system re-equilibration rather than contradictory responses. In Prairie contexts, where moisture pulses dominate process rates, NT effects are inherently nonlinear and strongly contingent on time since adoption. These conflicting findings across studies arise from interactions among soil texture, moisture regime, and duration of adoption, rather than inconsistency in microbial response mechanisms.

### 3.2. Effects of Crop Rotation on Soil Health Indicators

Table 2 synthesizes evidence showing that diversified crop rotations consistently shape Prairie soil physical structure, nutrient dynamics, and microbial functioning compared to monocultures. Long-term (12 yr) multi-crop rotations, particularly when integrated with no-till, enhance biologically mediated aggregation and pore formation, increasing soil porosity by 78% and aggregate stability by 5–20%, reflecting sustained inputs of microbial binding agents and root-derived biopolymers [57]. These structural gains translate into improved hydrological function, with 30–34% higher water retention [28], and 16–17% greater plant-available water capacity [57], alongside moderated thermal dynamics (warmer winters, cooler springs) driven by residue carryover and canopy effects that stabilize soil microclimate. Diversified rotations increased biomass inputs (+2710 kg dry matter ha^−1^), supplying substrates that fuel microbial growth, organic matter formation, and bioavailable N and K [57]. Inclusion of cover crops and legumes further tightens N cycling through off-season uptake and subsequent mineralization, and via symbiotic N_2_ fixation that benefits subsequent crops (e.g., canola, maize, wheat) [58,59]. Cumulative inputs of organic residues and microbial metabolites slightly acidify soils (0.2 to 0.4 pH units) while increasing cation exchange capacity, indicating enhanced chemical buffering and nutrient retention [60]. Collectively, these shifts are underpinned by a 50% rise in microbial diversity and selective enrichment of functionally important taxa (e.g., *Serratia*, *Pseudomonas*) [28]. Global meta-analysis studies support these trends, showing an increase of 13% microbial biomass carbon, 16% microbial biomass nitrogen, and 45% fungal biomass across temperate and semi-arid systems (<600 mm MAP) [61], elevated soil respiration rates (2–5 kg C ha^−1^ d^−1^) [60], pointing to a more metabolically active and resilient soil system under diversified rotations.

Crop rotation creates temporal variability in root traits, exudation, and residue inputs, expanding microbial niche diversity and enriching functional guilds (e.g., N-fixers, decomposers, P-solubilizers) [62,63,64]. Contrasting rooting depths redistribute nutrients and structure microbial activity along the soil profile. Deep-rooted crops (e.g., sunflower, safflower) stimulate subsoil processes, whereas shallow-rooted species (e.g., lentil, wheat) intensify surface turnover, collectively improving nutrient-use efficiency and reducing leaching [65,66]. Crops left a “legacy effect” on the preceding years’ crop root exudate profile, which modifies the chemical, physical, and biological properties of soil [36,58]. Thus, crop rotation promotes microbial resilience by continually resetting selective pressures and preventing functional stagnation of the soil microbiome.

Our synthesis found that the effects of crop rotation are governed by its structural complexity, specifically crop diversity, rooting depth variation, and continuity of organic inputs. Rotations that incorporate multiple crop types, legumes, and contrasting root architectures generate chemically diverse residues and spatially heterogeneous rhizospheres, which support broader microbial niches and functional diversity. This explains the more consistent increases in microbial biomass, fungal abundance, and water retention observed in diversified systems compared to monocultures or simple two-crop rotations. In particular, inclusion of legumes enhances nitrogen inputs and stimulates microbial activity, while deeper-rooted crops improve subsoil connectivity and resource capture. As a result, rotation-driven benefits emerge as an integration of aboveground diversity and belowground functional complementarity rather than a uniform “rotation effect.”

At the same time, Table 2 highlights that rotation effects are strongly context dependent. Biological and physical responses are generally more consistent, whereas chemical indicators show greater variability. Short-term or low-diversity rotations often produce limited or inconsistent responses, while long-term diversified systems show cumulative gains, underscoring the importance of temporal continuity. Notably, these benefits are accompanied by several trade-offs. For instance, legume inclusion can enhance nitrogen availability and microbial activity but may also increase the risk of nitrogen losses under wet conditions. Similarly, high-residue or diverse rotations can temporarily immobilize nutrients or delay early-season crop growth due to slower residue decomposition. Interactions with practices such as no-till further complicate outcomes, making it difficult to isolate rotation effects. Conflicting findings across studies, particularly in nutrient dynamics and early-season crop responses, are largely driven by differences in rotation complexity, soil fertility, and precipitation patterns.

Overall, Table 2 shows that the magnitude and direction of rotation effects are governed by structural complexity and temporal continuity, with diversified systems enhancing microbial niche partitioning and nutrient turnover efficiency, albeit with context-dependent trade-offs. Simplified rotations rarely capture the emergent properties observed in diversified systems. Reported inconsistencies arise from temporal decoupling between residue inputs and mineralization dynamics, often amplified under variable precipitation regimes.

**Table 2:** Effects of crop rotation on soil health indicators in Western Canada.

Crop Rotation Type	Soil Health Domain	Indicator	Effects	Experimental Design	Findings	Location of Study	References
**Multi-crop rotation**	**Physical**	Soil porosity	+78%	4 yr rotation; 7 Prairie sites	Root diversity increases macro- and microporosity	Prairie sites	[57]
**Diversified rotation under no-till**		Soil porosity	+35–75% (0–7.5 cm)	Diversified crops + NT	Microbial aggregation enhances pore structure	Lethbridge (AB); Swift Current and Scott (SK)	[57]
**Crop rotation (mixed annuals)**		Soil temperature	+2.5–5.7 °C (winter); −1.8–2.4 °C (spring)	Seasonal measurements	Residue and canopy effects buffer temperature	Ontario *	[67]
**Diversified rotation**		Aggregate stability	+5–20% (water-stable aggregates)	Surface soil; 4 yr study	Microbial binding agents stabilize aggregates	Prairie sites	[57]
**Diversified rotation**		Soil moisture retention	+0.01–0.03 m^3^ m^−3^ PAW; +8% microporosity; +16–17% PAWC	3 of 7 sites; 0–10 cm	Microbial aggregation improves water storage	Lethbridge (AB); Swift Current and Scott (SK)	[57]
**3–4 crop rotation (canola–wheat–pea/barley)**		Soil moisture retention	+30–34%	12 yr rotation	Diverse rooting systems enhance pore continuity	Lacombe (AB); Swift Current and Scott (SK)	[28]
**Soybean-based rotations (2–3 crops)**		Bulk density/resistance	↓ 0.3–0.5 MPa resistance; ~1 g cm^−3^ (*p* = 0.020)	Strongest under no-till	Improved structure offsets compaction	Ontario *	[68]
**Long vs. short rotation vs. perennial forage**	**Chemical**	Nutrient availability and SOC	Significant effects on NO_3_-N (*p* = 0.03), PMN (*p* = 0.02), SOC (*p* = 0.01)	4 yr vs. 2 yr vs. PER	Rotational legacy regulates microbial N cycling	MB; SK; AB	[28,69]
**Multi-crop rotation**		Biomass C input	+2710 kg DM ha^−1^	4 yr rotation	Increased residue inputs fuel microbes	Prairie sites	[57]
**Soybean-based rotations**		Soil pH and CEC	pH ↓ 0.2–0.4; CEC ↑ ~2 meq 100 g^−1^	Crop sequence effects	Organic inputs modify exchange capacity	Ridgetown (ON) *	[60]
**Soybean-based rotations**		Available P and K	+7–10 mg kg^−1^ (P); +19–30 mg kg^−1^ (K)	Rotational cropping	Microbial solubilization and residue cycling	Ridgetown (ON) *	[60]
**Long-term rotation**		Electrical conductivity	~2 dS m^−1^ (0.5 m depth)	Harvest-time moisture 20–30%	Improved water use reduces salt accumulation	Bow Island (AB)	[70]
**3–4 crop rotation**	**Biological**	Community composition	↑ diversity (~50%); Serratia (3.0%), Pseudomonas (3.3%)	12-yr rotation	Root exudate diversity selects functional taxa	AB; SK	[28]
**Rotation systems (meta-analysis)**		Microbial biomass	MBC +13%; MBN +16%; fungal biomass +45%	76 studies; <600 mm MAP	Rotation expands microbial C and N pools	Temperate and semi-arid	[61]
**2-crop rotation (corn-soybean)**		Soil respiration	2–5 kg C ha^−1^ d^−1^	Short-term rotation	Elevated microbial metabolic activity	Woodslee (ON) *	[61]

Footnotes: PAW = plant-available water; PAWC = plant-available water capacity; PMN = potentially mineralizable nitrogen; SOC = soil organic carbon; MBC = microbial biomass carbon; MBN = microbial biomass nitrogen; MAP = mean annual precipitation. Note: * Examples from outside Western Canada to provide a broader Canadian context. ↑ indicates an increase and ↓ indicates a decrease in the measured value compared to the control.

### 3.3. Cover Cropping

Table 3 synthesizes Canadian and Prairie studies on cover crops, highlighting their effects on soil microclimate, structure, nutrient cycling, and microbial activity during non-cash-crop periods. By modifying residue chemistry, root exudation, and habitat conditions, cover crops enhance microbial biomass, enzyme activity, and community structure while stabilizing the physical environment that governs microbial persistence. For instance, legume covers increased winter and spring soil temperatures (up to ~5.7 °C and 3.0 °C, respectively) in southwestern Ontario [71], while mixed legume covers (alfalfa-clover-hairy vetch) reduced bulk density by 3–4% in Québec, indicating improved pore connectivity [72]. Structural responses are species-dependent: in Saskatchewan, pea/lentil/Lathyrus covers modestly increased aggregate stability (2.3–4.1%), whereas sweet clover achieved substantially greater gains (65%) via enhanced microbial binding agents and fungal hyphal reinforcement [73]. Concurrently, cover crops regulate nutrient dynamics by altering substrate inputs and mineralization pathways; diversified species mixtures increased P (4.76%), K (6.67%), and Ca (5.51%) availability under low-input conditions, consistent with enhanced microbial mineralization [74]. However, effects on nitrate-N, potentially mineralizable N, and soil organic carbon are strongly contingent on rotation phase and system context [75]. These findings underscore that cover crops reconfigure microbially mediated nutrient retention and turnover based on system context (rotation phase, fertilization history, and residue management.

Biological indicators show that cover crops restructure microbial communities in ways that enhance resilience while introducing functional trade-offs. In Ontario, rye- and radish-based systems increased microbial biomass by 20.7–37% (up to 90 mg C g^−1^ TOC) but reduced alkaline phosphatase activity (−18% to −2.6%), indicating shifts in microbial C allocation and P-cycling enzyme expression [76]. Species identity further revealed significant increases in fungal and bacterial abundance (4.85% and 3.02%, *p* = 0.01). Moreover, enrichment of symbiotrophic fungi and nitrification-related bacteria under sorghum–sudangrass and buckwheat, demonstrating targeted selection of functional guilds linked to N cycling and symbiosis. System complexity amplifies these effects; for example, barley–rye mixtures increased aggregate stability by 10–32%, reflecting functional complementarity that broadens microbial niches and supports C stabilization [72,76,77]. Cover crops do not merely “increase microbes”; they selectively reassemble microbial guilds (symbiotrophs, nitrifiers, decomposers) and regulate enzyme-mediated functions, with statistically supported shifts depending on species and mixtures [28]. Interestingly, cover crops integrated with grazing (corn/grazing-soybean/grazing-cover crop/grazing) increased soil organic matter by 20–26% and improved macroporosity, infiltration rate, and total porosity over the long term [78].

We found that, compared with NT/RT and crop rotation, cover crops exhibit the greatest variability in both direction and magnitude of response, largely because their effects are governed by functional identity and management timing. Species composition drives distinct and divergent soil processes. Thus, the outcome pattern is not uniform, but a spectrum of responses linked to how well cover crop functional traits align with system needs. The inconsistencies across studies arise from interactions among species selection, moisture availability, termination timing, and background management systems. Notably, microbial activation and residue processing do not consistently translate into short-term agronomic benefits. In water-limited Prairie systems, cover crops can intensify soil moisture depletion, while high-residue inputs may temporarily immobilize nitrogen, leading to short-term nutrient constraints. These trade-offs explain divergent microbial and agronomic responses.

**Table 3:** Effects of cover crop functional groups on soil health indicators in Canada.

Cover Crop Functional Group	Soil Health Domain	Indicator	Effect	Experimental Design	Location of Study	References
**Legume cover crops** **(e.g., clover, alfalfa, vetch, sweet clover)**	**Physical**	Soil temperature	+2.5–5.7 °C (winter); +0.1–3.0 °C (spring) at 15 cm	Legume cover crops under seasonal cover	Southwestern Ontario *	[71]
		Bulk density	3–4% decrease	Mixed legume covers (alfalfa-clover-hairy vetch)	Québec *	[72]
		Aggregate stability	+2.3–4.1%; up to +65% (sweet clover)	Tilled and no-till comparisons	Saskatchewan	[72]
	**Biological**	Microbial diversity	↑ symbiotrophic fungi; ↑ nitrification-related bacteria	ITS and 16S amplicon sequencing	Prince Edward Island *	[79]
**Grass cover crops (e.g., rye, barley, oat, winter wheat)**	**Physical**	Bulk density	37–62% decrease (rye, sweet clover comparison)	No-till and rototill systems	Ontario *; Saskatchewan	[72]
		Soil temperature	+3 °C warmer (fall); −4 °C cooler (spring)	Seasonal monitoring	Eastern Canadian * Prairies	[72]
		Soil water content	+0.02–0.06 kg kg^−1^	Winter wheat cover	Westham Island, BC *	[80]
	**Chemical**	Soil organic C	+41% SOC	Rye cover under tillage	Ontario *	[72]
	**Biological**	Fungal and bacterial diversity	74–77% explained variability; ↑ Actinobacteria, Firmicutes, Ascomycota	Amplicon sequencing	Ontario *	[81]
**Brassica cover crops (e.g., oilseed radish, mustard)**	**Chemical**	Nutrient availability	P, K, Ca ↑ by 4.76–6.67% (*p* ≤ 0.05)	No synthetic fertilizer	Prince Edward Island *	[74]
		Nitrate-N and PMN	Significant effects (*p* ≤ 0.03)	Rotation and cover crop phases	MB; SK; AB	[75]
	**Biological**	Microbial biomass and enzymes	↑ 90 mg C g^−1^ TOC; ↑ alkaline phosphatase	RAD and RAD + rye systems	Ontario *	[76]
**Mixed cover crops (legume + grass/grass + brassica)**	**Physical**	Aggregate stability	+10–32%	Barley-rye mixtures	British Columbia *	[72]
	**Chemical**	SOC and nutrient pools	SOC: No CC = 19.34; Rye = 26.01; RAD = 27.19; RAD + Rye = 26.42	Mixed covers	Prairie sites	[76]
	**Biological**	Microbial biomass and diversity	Microbial biomass ↑ 20.7–37%	Mixed covers	Ontario *	[76]
**Cover crops with grazing.**	**Physical and Chemical**	SOM, porosity, infiltration	SOM ↑ 20–26%; ↑ macroporosity and infiltration	Integrated crop-livestock systems	Prairie sites	[78]

Footnotes: SOC = soil organic carbon; SOM = soil organic matter; PMN = potentially mineralizable nitrogen; RAD = oilseed radish. Note: * Examples from outside Western Canada to provide a broader Canadian context. ↑ indicates an increase in the measured value compared to the control.

As reflected in Table 3, cover crop responses are highly variable and driven by species identity, management timing, and moisture conditions, highlighting their role as adaptive, rather than universally beneficial, microbial interventions. Overall, cover crops function as management-sensitive practices whose effects are conditionally expressed. Their performance in Prairie systems reflects tight coupling between functional traits and water limitation, requiring precise alignment of species choice and management timing with climatic windows. This explains why conflicting outcomes are frequently reported, where strong microbial responses coexist with neutral or negative yield responses under water-limited conditions.

### 3.4. Effects of Organic Amendments on Microbial Dynamics and Soil Health

The directional patterns synthesized and summarized in Supplementary Tables S2 and S3 indicate that organic amendments consistently promote microbially mediated carbon and nutrient cycling, with response magnitude governed by input quality and decomposability. Manure and compost consistently drive the strongest increases in microbial biomass, diversity, and SOC, translating into the most reliable yield gains (approximately 27% and up to 49% under manure amendments); similar patterns of yield and disease benefits are reported in Prairie studies and biorational management work [82]. In contrast, crop residues primarily stimulate carbon-degrading enzymes and microbial biomass, but are frequently coupled with transient N immobilization, leading to weaker or delayed yield responses. Biochar mainly enhances stable SOC and microbial habitat structure; however, its effects on nutrient cycling and yield are variable unless co-applied with nutrient-rich inputs [83]. Overall, these contrasts indicate that amendment type governs whether microbial responses prioritize growth, enzyme production, or habitat stabilization, reflecting underlying substrate chemistry. Statistically significant gains in microbial biomass (up to 51%), enzyme activity, and carbon stabilization under organic inputs reflect improved nutrient-use efficiency and sustained yield benefits (up to 27%), particularly in low-SOC, semi-arid Prairie soils [82,84].

Supplementary Table S3 demonstrates that responses to organic amendments are primarily structured by substrate quality and nutrient stoichiometry rather than carbon addition alone. Labile, nutrient-rich inputs (livestock and green manures) consistently drive strong increases in microbial biomass, enzyme activity, and nutrient cycling, resulting in robust yield responses. Compost shows similar but more moderate effects, reflecting its intermediate decomposability and role in sustained SOC accumulation. In contrast, crop residues stimulate microbial activity and carbon-degrading enzymes but are often associated with temporary N immobilization and weaker short-term productivity gains, while biochar mainly enhances carbon persistence and microbial habitat with limited direct effects on nutrient turnover. Collectively, these responses indicate distinct microbial strategies and soil functions emerge across amendment types. Variability across studies reflects differences in substrate quality, C:N ratios, and environmental conditions rather than inconsistency in microbial processes.

Observed variability arises from differences in decomposability (labile vs. recalcitrant carbon), C: N ratios, and the functional role of each amendment. Nutrient-rich inputs promote rapid microbial growth and turnover, whereas high C: N residues shift microbial activity toward carbon acquisition, constraining immediate nutrient availability. Biochar’s inconsistent responses reflect its structural role in improving retention and habitat rather than directly supplying nutrients. These differences explain why similar amendments produce divergent outcomes across systems. Synthesis of Supplementary Tables S2 and S3 indicates that organic amendment responses are structured by substrate quality and nutrient stoichiometry, reflecting a trade-off between rapid nutrient cycling and long-term carbon stabilization.

### 3.5. Impact of Regenerative Agriculture (RA) Practices on Crop Yield and Long-Term Sustainability in Western Canada

Table 4 demonstrates that RA practices enhance yield stability and resource-use efficiency over time, with benefits emerging primarily through microbial-mediated improvements in soil function rather than immediate yield gains. In Western Canada, no-till and reduced-till systems consistently increased wheat yields by 10–147% over 4–10 years, with the largest gains observed during severe drought years [85]. Studies indicate cumulative annual yield gains up to 10% across wheat, canola, and pulses under long-term no-till, reflecting progressive improvements in soil structure, organic carbon, and microbial function [86]. In contrast, short-term or site-specific studies show more variable responses, highlighting the importance of the temporal scale under high interannual climate variability [47,87]. Crop rotation emerged as one of the most reliable RA strategies, particularly with pulse inclusion; long-term rotations (≥12–30 years) increased grain yield by 14–38% and protein yield up to 66%, especially in semi-arid, low-fertility systems, by enhancing biological nitrogen fixation and nutrient cycling [77]. Multi-site Prairie studies also reported moderate but consistent cereal yield gains (0.1–0.5 t ha^−1^) under diversified rotations, buffering against climatic stress rather than maximizing yields [57]. Diversified rotations improved soil microbial biomass, functional diversity, and nitrogen-use efficiency, translating into more resilient yields [28,61]. Cover crops and organic amendments delivered context-dependent benefits: cover crops occasionally caused small yield penalties under drought or suboptimal termination [59], whereas long-term cover crop mixtures in Ontario increased corn yields by 38–59 bu ac^−1^ over 14 years, especially during mid-season droughts. A 25-year stewardship program in southwestern British Columbia enhanced SOC stocks by 71% and soil workability by 25% [87]. Organic amendments increased yields up to 49%, though effects were dependent on amendment type, rate, soil, and climate [82,83]. Collectively, Table 4 shows that stacking and sustaining RA practices stabilize yields, reduce external inputs, and reinforce agroecosystem resilience, with microbial regeneration as a key mechanistic link. Table 4 indicates that the most consistent pattern of RA practices in Western Canada is not an immediate increase in crop yield, but a progressive stabilization of productivity under variable climatic conditions. Practices such as NT/RT, diversified rotations, and cover crops collectively enhance soil moisture retention, structural stability, and microbial activity, which buffer crops against drought and temperature variability, key constraints in Prairie systems. Yield responses therefore tend to be neutral or modest in the short term, particularly during early adoption phases, but become more stable and, in some cases, positive over time as soil structure and biological functioning improve. This pattern suggests that RA practices primarily act through risk reduction and resilience building rather than direct yield maximization, with benefits emerging as system-level properties rather than immediate outputs.

Yield variability across RA systems is best interpreted through the lens of resilience rather than productivity maximization, with microbial-mediated improvements enhancing stability under climatic stress rather than absolute yield potential. In moisture-limited environments, cover crops may temporarily compete for water, while in wetter systems, they enhance nutrient cycling and yield stability. No-till (NT) has been reported to increase grain yield by up to 147% compared with conventional tillage under heat and water stress, largely due to improved soil moisture retention and reduced root stress during grain filling [80]. Reported contradictions stem largely from differences in study duration and precipitation regimes, which determine whether biological gains offset resource limitations. Early NT adoption may show yield penalties from compaction or nutrient stratification, whereas long-term NT improves pore structure and microbial-driven nutrient supply. Residue management and fertilization practices further contribute to inconsistent outcomes. Trade-offs are therefore inherent: gains in soil health and resilience may coincide with delayed yield responses or increased management complexity. In semi-arid Prairie systems, the primary agronomic value of RA lies in buffering interannual variability rather than consistently increasing mean yields. Conflicting yield responses across studies primarily reflect differences in climatic stress, soil conditions, and duration of practice adoption rather than inconsistency in regenerative mechanisms.

### 3.6. Effects of RA Practices on Weed, Insect, and Pathogen Suppression

Multiple long-term field studies revealed that RA practices can reduce weed emergence and soilborne disease pressure across Prairie systems, primarily through microbial competition, antagonism, and network stabilization. These effects strengthen with system duration, diversity, and integration, rather than single-practice implementation. A 4-year cereal-pulse-cover crop rotation at Lethbridge, AB, significantly reduced weed germination (*Kochia scoparia*, *Amaranthus retroflexus*) and suppressed root rot pathogens (*Fusarium graminearum*, *Rhizoctonia solani*), driven by allelopathic residues and increased antagonistic microbes (*Pseudomonas, Bacillus*) and AMF colonization [89]. Microbial-mediated mechanisms for suppressing pathogens, pests, and weeds are summarized in Supplementary Table S4. Long-term no-till systems evidenced cumulative and stabilizing suppression effects. A 12-year zero-till rotation in Swift Current (SK) reduced annual weeds (*Avena fatua, Setaria viridis*) and pathogen inoculum (*Fusarium* spp., *Gaeumannomyces graminis*), associated with preserved fungal hyphae, actinomycetes enrichment, and weed-suppressive *Pseudomonas fluorescens* [90]. Notably, suppression strengthened with duration, underscoring that soil microbial network stabilization, rather than immediate disturbance reduction. A 6-year high-residue wheat-canola rotation in Manitoba delayed spring weed emergence and reduced early-season foliar diseases via saprophytic fungi (*Streptomyces*) and phenolic compounds [91], indicating residue quantity and persistence regulate microbial antagonism. We found integrated systems intensify suppression through trophic and microbial feedback. In Carman, Manitoba, a 3-year cover crop-livestock integration reduced perennial weeds (*Cirsium arvense*) and clubroot (*Plasmodiophora brassicae*), linked to manure-derived microbial inputs and induced systemic resistance [92]. An 8-year high-diversity rotation at Lacombe, AB, significantly reduced root disease buildup (*Fusarium avenaceum, Pythium* spp.) through diversified root exudation and increased AMF colonization [93].

Taken together, Table 5 reveals that across Prairie systems, pest and pathogen suppression consistently increases with system diversification, residue continuity, and time, indicating that microbial network complexity is the primary driver of biological control. The magnitude of suppression varies with rotation length, residue levels, moisture conditions, and integration (e.g., livestock), reflecting context-dependent microbial assembly and activity. Short-term or single-practice systems show weaker or inconsistent effects. These microbe-mediated effects reduce reliance on synthetic inputs while reinforcing soil health and yield stability, linking suppression outcomes (Table 5) with microbial processes (Table 1, Table 2 and Table 3) and yield responses (Table 4). However, enhanced microbial suppression may involve slower early-season crop emergence (due to residue effects), transient nutrient immobilization, or delayed response timelines, highlighting that biological control builds gradually and may not immediately replace chemical interventions.

Collectively, Table 5 indicates that RA-driven microbial diversification enhances biocontrol potential, although outcomes vary with system complexity, environmental conditions, and management intensity. Biocontrol outcomes are governed by the assembly and persistence of complex microbial networks, which require time and resource continuity to develop functional redundancy and antagonistic capacity. Inconsistent suppression in short-term studies reflects incomplete community maturation rather than failure of RA principles. Effective disease and pest regulation, therefore, emerges as a long-term, system-level property contingent on diversity, residue management, and moisture regimes. Inconsistent suppression observed in short-term studies reflects incomplete microbial network development rather than failure of regenerative practices.

### 3.7. Microbial Contributions to Input Reduction and Agronomic Efficiency

In Western Canada, agrochemicals are widely used to sustain crop productivity by improving nutrition and suppressing weeds, pests, and diseases. Herbicides (glyphosate, 2,4-D, and dicamba) dominate for weed management [94], while fungicides (azoxystrobin and propiconazole) target diseases such as sclerotinia, fusarium head blight, and rusts in crops [95,96,97], and insecticides (e.g., lambda-cyhalothrin, deltamethrin) manage key pests [98]. Synthetic fertilizers (N, P, K, S) remain essential for yield optimization [99] but may bypass or disrupt microbially mediated nutrient cycling and pest regulation pathways. Table 6 integrates evidence on how RA practices reduce reliance on these synthetic fertilizers and pesticides by enhancing microbial nutrient cycling, biological nitrogen fixation, residual N carryover, and pest regulation. Long-term no-till combined with diversified rotations reduced synthetic nitrogen use by up to 73% and herbicide application by 42%, driven by soil organic matter accumulation, improved N cycling, and reduced disease pressure [100,101]. Pulse-based systems are central to nitrogen reduction, with residual N credits range from 10–60 kg N ha^−1^. Intercropping systems (e.g., pea–oat, pea–canola) further reduce fertilizer demand by 5–25 kg N ha^−1^ and lower fungicide and insecticide use [50,102]. Intercropping (e.g., pea-oat, pea-canola) while lowering reduced mineral N demand by 5–25 kg N·ha^−1^ while lowering fungicide and insecticide use across sites in Alberta, Saskatchewan, and Manitoba [103]. At the system scale, integrated strategies (crop rotation, intercropping, IWM, precision N) reduce fertilizer inputs by 10–60 kg N ha^−1^ and pesticide use by ~20–60% without compromising yield stability. Rhizobia inoculation substitutes up to 30% of synthetic N inputs, though responses vary with soil and climate [104,105].

This synthesis clearly indicates that RA practices consistently reduce dependence on synthetic nitrogen and chemical pesticides, but the magnitude and reliability of these reductions are strongly conditioned by practice integration, duration, and crop context. Input reduction consistently follows enhanced microbial functioning, particularly through biological N fixation, improved nutrient-use efficiency, and microbially mediated pest suppression, indicating that reduced inputs are an emergent property of biologically active soils. The extent of input reduction depends on crop type (especially pulse inclusion), system integration, climate variability, and duration of RA adoption, with the strongest effects observed in long-term, diversified systems. Reduced synthetic inputs may involve short-term yield variability, dependence on biological N timing, and increased management complexity (e.g., rotation design, precision practices), indicating that efficiency gains require system-level coordination rather than simple input withdrawal. As summarized in Table 6, reductions in external inputs are primarily achieved through enhanced microbial nutrient cycling and biological regulation, with outcomes dependent on system integration and long-term adoption.

## 4. Discussion

### 4.1. Microbial Restoration as the Integrating Mechanism of Regenerative Agriculture

Figure 1 conceptualizes RA as a process that rebuilds soil systems primarily through biologically mediated mechanisms than through the simple substitution of conventional inputs. Decades of intensive tillage, monocropping, summer fallow, and heavy agrochemical use in the Canadian Prairies have progressively damaged microbial habitats, disrupted fungal networks, and narrowed functional genetic diversity, contributing to poor soil structure, inefficient nutrient cycling, and inconsistent crop performance [5]. Evidence synthesized across this review and in comparable global assessments indicates that RA practices such as no tillage, diversified rotations, cover cropping, organic amendments, and crop-livestock integration can improve microbial habitat continuity, increase carbon inputs to the rhizosphere, and enhance functional redundancy within soil microbial communities, although responses remain context dependent [74,107,108]. RA outcomes in prairie systems can be conceptualized as a stimulation–response–outcome cascade, in which management alters disturbance and resource regimes, triggers microbial responses that reorganize soil function and associated ecosystem services [2]. This synthesis reframes regenerative agriculture not as a discrete set of practices, but as a strategy for restoring soil as a self-organizing biological system, with microbial network integrity acting as a key, though not exclusive, control point for soil process and agronomic outcomes.

### 4.2. Mechanistic Framework Linking Regenerative Practices to Soil Microbial Function and Agronomic Outcomes

**Figure 2:**
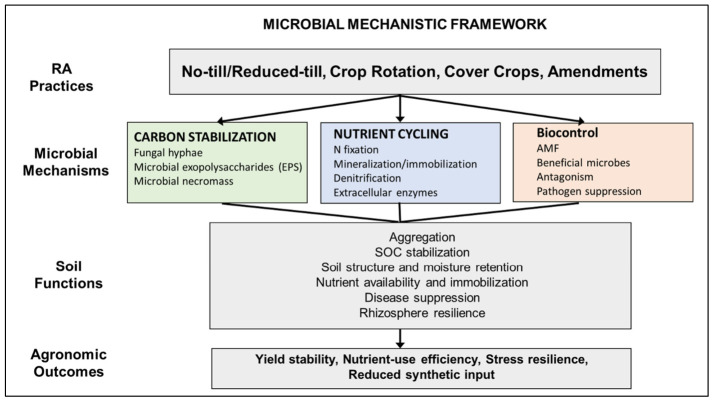
Flowchart of regenerative agriculture (RA) pathways linking management practices to microbial processes, soil functions, and agronomic outcomes, highlighting carbon stabilization, nutrient cycling, and biocontrol as key drivers of yield stability and system sustainability.

RA influences agroecosystem performance through microbially mediated processes that integrate plant inputs, soil properties, and environmental conditions. Rather than directly driving productivity, RA practices reorganize the soil biological system, with microorganisms functioning as key agents that translate management interventions into soil functions and agronomic outcomes. This mechanistic linkage is illustrated in Figure 2, which integrates management practices with microbial pathways and ecosystem outcomes. Reduced disturbance and continuous organic inputs enhance fungal networks, microbial exopolysaccharides, and necromass, which promote soil aggregation and stabilize soil organic carbon. The improved moisture retention and nutrient immobilization manifest agronomically as enhanced yield stability and drought resilience. Findings from Table 1 and Table 3 reflect the gradual nature of biologically driven structural recovery. Diversified crop rotations and biologically active systems shift microbial nutrient transformations such as nitrogen fixation, mineralization, and denitrification via functional groups, enhancing nutrient availability, retention, and use efficiency while reducing external inputs. As shown in Table 2 and Supplementary Tables S2 and S3, these responses are strongly influenced by rotation complexity, residue quality, and system integration, with more diverse systems exhibiting greater functional redundancy and nutrient buffering capacity. Higher plant diversity and lower disturbance foster beneficial microbes, including arbuscular mycorrhizal fungi and rhizosphere bacteria, suppressing pathogens, strengthening rhizosphere resilience, and supporting crop health, thus lowering chemical protection needs. These effects are closely linked to system diversity and continuity of plant inputs, with integrated systems showing the most consistent responses.

At the same time, the framework in Figure 2 highlights that outcomes are inherently context-dependent. Variability observed across Table 1, Table 2, Table 3, Table 4 and Table 5 can be attributed to differences in time since adoption, soil depth, soil type, and moisture regime. For example, short-term increases in surface bulk density under no-till may temporarily constrain physical function, while moisture-limited conditions can shift nutrient cycling toward less favorable pathways [38,39]. Similarly, cover crops may enhance microbial activity yet introduce trade-offs such as water competition or delayed nutrient release [54,71]. These dynamics explain why agronomic outcomes, summarized in Table 4 and Table 5, are more consistently expressed as improvements in yield stability, resource-use efficiency, and long-term sustainability rather than immediate yield gains.

### 4.3. Microbial Restoration as the Foundation for Pest Suppression and Input Reduction

Pest suppression and input reduction in regenerative systems are best viewed as emergent ecosystem properties of restored soil microbiomes, rather than as direct outcomes of any single agronomic intervention. The patterns of soil biological recovery summarized in Table 1, Table 2 and Table 3 and Supplementary Tables S2 and S3 provide a mechanistic insight for the consistent reductions in weeds, plant pathogens, and chemical inputs observed in the studies documented in Table 5 and Table 6. Across Prairie field studies, weed suppression, disease regulation, and lower pesticide dependence emerge not from any single management action but from the collective effects of microbial niche competition, antagonistic interactions, and stabilization of soil microbial networks. Long-term reduced tillage and diversified crop rotations selectively enrich populations of Pseudomonas, Bacillus, Streptomyces, and arbuscular mycorrhizal fungi (AMF), providing a plausible biological explanation for sustained suppression of *Fusarium*, *Rhizoctonia*, and weed establishment over time [93,114]. The inclusion of cover crops and organic amendments further amplifies enzyme activity and accelerates decomposition of allelopathic residues, reinforcing microbial dominance within the rhizosphere and strengthening biotic control of pests and pathogens [115]. Importantly, these microbe-mediated processes translate into quantifiable reductions in synthetic inputs. As summarized in Table 6, residual nitrogen contributions from pulse-based crop rotations, symbiotic N fixation by rhizobia, and improved microbial nitrogen-use efficiency collectively reduce fertilizer requirements by approximately 10–60 kg N ha^−1^, while integrated weed management, cultivar selection, and threshold-based decision frameworks decrease herbicide and insecticide use by roughly 20–60% without compromising yield stability [50,103,116,117]. Together, these findings support the key premise of Figure 1: in prairie agroecosystems, reduced reliance on external input emerges from biologically functional, microbially regulated soils, rather than serving as a primary starting point for regenerative management.

### 4.4. Yield Stability and Prairie-Global Convergence Under Climatic Constraint

The yield responses synthesized in Table 4 broadly mirror patterns reported in the global regenerative agriculture (RA) literature, but they are more tightly constrained by the distinctive climate of the Canadian prairies. Globally, RA is associated with consistent increases in soil organic carbon, microbial biomass, and ecosystem resilience, while yield outcomes remain context dependent, frequently neutral to positive in rainfed and biologically diversified systems, yet more variable where water or nutrient competition is poorly managed [118,119]. Prairie studies align closely with this global pattern: long-term no-till and diversified rotations reliably improve yield stability and drought resilience, whereas short-term yield penalties following cover crop adoption occasionally occur in short-season, moisture-limited conditions [59]. Crucially, these trade-offs do not imply a failure of RA principles but instead reflect temporal and climatic modulation of microbial-mediated feedbacks. Yield responses therefore appear to follow a threshold pattern, in which early biological recovery improves resilience first, while consistent yield gains emerge only after cumulative improvements in aggregation, SOC stabilization, and nutrient buffering are achieved. Low soil temperature, episodic drought, and slower residue decomposition can delay the conversion of microbial recovery into yield gains. Figure 1 captures this dynamic by emphasizing time, practice stacking, and context specificity as prerequisites for interpreting agronomic outcomes. Where RA practices are implemented as integrated, tailored to local climate, soil conditions, and production constraints, yield benefits tend to accumulate and interannual variability declines, consistent with long-term global findings [82].

### 4.5. Metagenomic Evidence of Functional Resilience of Prairie Soil Microbiomes Under RA

In the Western Canadian Prairies, RA practices primarily influence soil microbiomes through functional reprogramming rather than large-scale compositional change, a pattern best resolved through metagenomics-informed and gene-centric approaches. Across prairie agroecosystems, long-term no-till systems in Alberta and Saskatchewan show relatively stable microbial community structures but increased biomass, stratification, and residue-associated activity, indicating that reduced disturbance enhances microbial efficiency rather than driving major genomic turnover [48]. Metagenomic and functional gene evidence further suggest that RA practices selectively enrich pathways linked to carbon stabilization, nitrogen cycling, and substrate utilization efficiency, consistent with broader observations of metabolic adaptation under agricultural management [59,120].

A recurring pattern in prairie soils is strong functional redundancy, where microbial gene pools remain relatively conserved despite environmental or management variation, buffering ecosystem processes against taxonomic shifts [121]. Although direct shotgun metagenomic studies remain limited, available prairie evidence shows that edaphic gradients, particularly soil depth, exert stronger control over functional gene distribution than management practices, especially for nitrogen cycling and stress-response pathways [122,123]. Within this framework, RA practices such as no-till, crop rotation, and cover cropping appear to influence soil functioning primarily through selective activation of microbial metabolic pathways rather than structural reorganization of microbiomes, with observed increases in enzyme activity and nutrient cycling potential constrained by semi-arid climatic conditions [36,48,79,124]. These findings indicate that RA impacts in prairie agroecosystems are best understood as functional optimization within a resilient and environmentally constrained microbial gene pool, where microbial processes respond more strongly than community structure. This highlights a critical need for integrating shotgun metagenomics with long-term field experiments to directly link microbial functional potential with agronomic outcomes under semi-arid prairie conditions.

### 4.6. System-Level Implications and Global Relevance of Prairie Evidence

Synthesizing evidence from Prairie field experiments (Table 1, Table 2, Table 3, Table 4, Table 5 and Table 6) and global meta-analyses demonstrated that the core biological mechanisms underpinning regenerative agriculture (RA) are broadly conserved across agroecosystems, including enhanced microbial biomass, restored fungal: bacterial balance, strengthened AMF networks, and improved carbon stabilization pathways. However, the magnitude, rate, and reliability of outcomes vary substantially, depending on alignment with local climate, soil texture, baseline soil organic carbon, and water regimes. Recent global syntheses consistently report that integrated RA systems restore microbial network stability, increase microbial carbon-use efficiency, and reinforce biologically mediated pest regulation, thereby enhancing yield resilience under climate stress [22,35]. Prairie datasets mirror these global patterns but further illustrate that soil health recovery precedes yield gains, particularly under semi-arid and short-season constraints. For example, long-term no-till and diversified rotations in Saskatchewan and Alberta increased SOC stocks, microbial biomass, and enzyme activity (Table 1 and Table 2), while yield stabilization became evident only after multi-year system restructuring (Table 4). Thus, prairie evidence confirms that regenerative transitions operate through cumulative microbial feedbacks rather than single practice effects, reinforcing the global conclusion that system integration, not isolated adoption, determines resilience trajectories.

Importantly, the distinction between global and Western Canadian RA outcomes lies not in the underlying microbial processes, which remain mechanistically similar, but in the temporal dynamics through which biological recovery translates into consistent yield and input-efficiency benefits under climatic constraint. In moisture-limited Prairie systems, low soil temperatures and episodic drought can delay residue decomposition, microbial turnover, and nutrient mineralization, slowing the agronomic expression of microbial gains. However, once SOC thresholds and microbial network stability are achieved, Prairie systems exhibit pronounced improvements in drought buffering, disease suppression, and reduced synthetic N and pesticide reliance (Table 5 and Table 6), aligning closely with global resilience patterns reported in semi-arid and temperate systems [80,85,117]. Taken together, these findings position the Canadian Prairies as a critical stress-test for regenerative systems globally, a region where climatic limitation sharpens the signal of microbial functionality, thereby clarifying how carefully calibrated, multi-year regenerative strategies can sustain productivity, suppress pests, and reduce chemical dependence under environmental constraint.

Despite growing evidence supporting microbiome-informed regenerative practices, several barriers constrain their large-scale adoption in Prairie agroecosystems. Economically, producers face transition costs and uncertain short-term returns, particularly under variable climatic conditions [14]. Technically, translating microbial indicators into actionable management strategies remains limited by methodological complexity and a lack of standardized thresholds. Knowledge-based barriers further limit adoption, including insufficient region-specific guidance linking microbial metrics to agronomic performance [14,125]. These challenges are amplified by the context-dependent nature of microbial responses to climate, soil, and management [52,59]. Addressing these constraints will require integrated frameworks that couple microbial diagnostics with decision-support tools, economic incentives, and locally validated management strategies.

## 5. Conclusions

### 5.1. Well-Established Evidence

This synthesis provides strong evidence that RA enhances agroecosystem resilience in the Canadian Prairies by re-establishing soil as a biologically complex and functionally adaptive system, consistent with the framework illustrated in Figure 1. RA in the Western Canadian Prairies shows clear, well-established benefits for soil biological functioning. Across long-term studies, increases in microbial biomass, diversity, enzymatic activity, and mycorrhizal abundance consistently translate into improved soil organic carbon, aggregation, water retention, and nutrient-use efficiency. These outcomes are robust across soil zones and represent the most certain and reproducible effects of RA.

### 5.2. Context Dependency and Remaining Uncertainties

Responses related to cover crop performance and short-term yield effects remain uncertain and strongly context-dependent, driven by soil type, moisture availability, and management timing. Many reported inconsistencies reflect transitional system dynamics and temporal adjustment processes, as well as unresolved links between microbial composition and functional outcomes. Similarly, microbial contributions to N_2_O emissions, pest suppression, and deep-soil carbon stabilization remain incompletely resolved, particularly under the Prairie’s extreme moisture variability. These patterns indicate that observed variability across studies reflects interactions among climate, soil conditions, and duration of adoption, rather than inconsistency in regenerative mechanisms.

### 5.3. Research Gaps and Future Priorities

Major knowledge gaps constrain advancing microbiome-informed RA in Prairie systems: limited mechanistic understanding linking microbial network structure to ecosystem functions; insufficient quantification of trade-offs, particularly N_2_O emissions, moisture-driven metabolic shifts, and cover crop competition; and a lack of translation of microbial indicators into actionable thresholds or predictive tools for management. Future priorities should focus on long-term, system-level experiments integrating metagenomics, enzyme kinetics, carbon fluxes, and microbial network modeling with agronomic outcomes. Particular emphasis is needed on identifying microbial thresholds linked to resilience, quantifying moisture-driven trade-offs, and evaluating practice stacking under Prairie climatic constraints.

### 5.4. Toward Microbiome-Informed Regenerative Systems

Advancing these priorities will enable microbiome-informed, climate-adaptive frameworks capable of guiding climate-adaptive regenerative transitions across the Western Canadian Prairies. In this context, soil microbial communities should be viewed not only as indicators but as active regulators linking management practices to soil functions and agronomic outcomes, providing a foundation for sustainable and resilient agroecosystem management under increasing climate variability.

## Figures and Tables

**Table 1:** Effects of no-till (NT) and reduced tillage (RT) on soil health indicators in Western Canada.

Soil Health Domain	Indicator	Effect of NT/RT Relative to CT	Experimental Design	Location of Study	Findings	References
**Physical**	Soil moisture retention	+3.5–5.6% VWC; +3 ± 0.02 m^−3^ VWC	Long-term NT; residue retained; some NT + cover crop systems; Crop rotation	Saskatchewan Alberta	Residue reduces evaporation; improved pore continuity and snow trapping	[38,39]
	Soil temperature	−0.8 to −9.9 °C (summer); warmer in winter	Residue-covered NT soils; surface measurements	Ontario * Prairies	Residue buffers diurnal and seasonal thermal extremes	[40]
	Aggregate stability	+7–38% macroaggregates (>12.7 mm); fewer fine aggregates	NT vs. CT; straw retained vs. removed; multi-year trials	Saskatchewan	Fungal hyphae and microbial binding agents were preserved	[41,42]
	Bulk density	Slightly higher at the surface (0–10 cm); structure improves over time	Long-term NT (0–15 cm); corn-soy and cereal systems	Ontario * Prairies	Reduced disturbance increases surface packing; SOC offsets compaction	[43,44]
**Chemical**	Soil Organic Carbon (SOC)	Across long- term NT studies over 11–47 yrs increased up to 15.6 kg C ha^−1^ yr^−1^	NT with reduced summer fallow; continuous and diversified rotations	Saskatchewan; Alberta	Slower residue decomposition; enhanced C stabilization	[53]
	Nitrogen availability	+13–47% crop N uptake	NT vs. CT; straw retained (S) vs. removed (NS); 0–120 kg N ha^−1^	Alberta; Saskatchewan	Enhanced microbial mineralization and N retention	[46]
	Soil pH stratification	Lower pH at 10–30 cm	Long-term NT; minimal soil mixing	Québec; Ontario *	Reduced vertical redistribution of acidity	[47]
**Biological**	Microbial biomass C	+40–86% (0–5 cm)	Long-term NT (>25 yr); Prairie soil zones	Prairie sites	Habitat stability and increased organic inputs	[48,49]
	Enzyme activity	↑ β-glucosidase, cellulase, xylanase, phosphatase	NT vs. CT across multiple Prairie sites	Alberta Manitoba Saskatchewan	Accelerated C cycling and nutrient turnover	[51,54]
	AMF biomass	+32–60%	NT surface soils; residue retention	Saskatchewan	Preservation of mycorrhizal networks	[49]
	Functional N cycling genes	↑ *nifH*, *nirK*; altered (*nirK* + *nirS*)/*nosZ*	NT surface soils; moisture-responsive systems	Prairie sites	Enhanced N cycling with potential N_2_O trade-offs	[52]

Footnotes: CT = conventional tillage; NT = no-till; RT = reduced tillage; SOC = soil organic carbon; AMF = arbuscular mycorrhizal fungi; VWC = volumetric water content. Note: * Examples from outside Western Canada to provide a broader Canadian context. ↑ indicates an increase in the measured value compared to the control.

**Table 4:** The impact of RA practices on crop yield and sustainability indicators in Western Canada.

RA Practices	Crop System	Yield Effect	Duration	Location	Experimental Condition	Climate Stress Context	Sustainability Signal	Key References
**No till/** **Reduced till**	Wheat	+10–147%	1–10 yr	AB, SK (multiple sites)	Continuous wheat or combined with crop rotation (CR)	Drought-prone Prairies	Yield stability ↑; SOC accumulation	[85]
	Wheat (multi crop synthesis)	+7% yr^−1^	Long-term	Western and Eastern Canada *	NT adoption across cropping systems	Drought	Climate resilience ↑	[88]
	Canola	+10% yr^−1^	Long-term	Canada	NT systems	Variable Rainfall	Input efficiency ↑	[88]
	Pulses	+9% yr^−1^	Long-term	Canada	NT systems	Semi-arid	N-use efficiency ↑	[88]
	Mixed cereals	↔/slight ↑	24 yr	Québec *	Long-term NT vs. CT	Climate variability	Yield resilience ↑	[47]
**Crop rotation**	Canola–wheat–pea/barley	+421 kg ha^−1^	12 yr	AB, SK	Diversified rotation	Variable precipitation	Stability ↑	[28]
	Wheat and cereals	+0.1–0.5 t ha^−1^	4 yr	AB, SK	Diversified rotation	Prairie drought	Yield buffering	[57]
	Wheat–canola–wheat–pea	+14–38%	>30 yr	SK	Long-term rotation	Semi-arid	NUE ↑; SOC ↑	[77]
	Wheat–canola–wheat-pea	Grain +38%; protein +66%	12 yr	SK	Pulse inclusion	Drought	Nutritional resilience ↑	[77]
**Cover crops**	Mixed cash crops	Slight ↓ (dry yrs)	1–3 yr	SK	Legume and grass covers	Low rainfall	Risk-reward trade-off	[59]
	Grain corn	+38–59 bu ac^−1^	14 yr	ON *	Legume and non-legume mixes	Mid-season drought	Long-term gain ↑	Experiment by Grain Farmers of Ontario (GFO) (2007–2021)
	Soybean–wheat–corn (organic)	Soybean +5–10%; wheat 8–9%	2–3 yr	ON *	Legume covers: organic	Variable seasons	System resilience ↑	Experiment by Grain Farmers of Ontario (GFO) (2022)
	Wheat, barley	+27–49%	Multi yr	AB, SK	Manure, compost, biochar	Semi-arid	Biological fertility ↑	[82,83].

Footnotes: SOC = soil organic carbon; NUE = nitrogen-use efficiency; NT = no-till; CT = conventional tillage; CR = crop rotation. * Examples from outside Western Canada to provide a broader Canadian context. Yield response expressed relative to conventional or control systems unless stated otherwise. ↔ indicates no statistically significant difference. ↑ indicates an increase and ↓ indicates a decrease in the measured value compared to the control.

**Table 5:** Effects of RA practices on weed, insect, and pathogen suppression in Western Canada.

Regenerative Practice	Weed Suppression (Directional)	Insect/Disease Suppression (Directional)	Key Target Species	Dominant Microbial Mechanisms	Experimental Location and Duration	Reference
**Cover crops**	↓ weed emergence	↓ root rot incidence	*Kochia scoparia*, *Amaranthus retroflexus*; *Fusarium graminearum*, *Rhizoctonia solani*	Allelopathic phenolics; ***Pseudomonas***, ***Bacillus*** antibiotics; AMF-mediated nutrient competition	Lethbridge, AB; 4 yr cereal–pulse–cover crop rotation	[89]
**No-till/minimal disturbance**	↓ annual weeds (long-term)	↓ soilborne pathogens	*Avena fatua*, *Setaria viridis*; *Fusarium* spp., *Gaeumannomyces graminis*	Preserved AMF hyphal networks; antagonistic actinomycetes; weed-suppressive ***Pseudomonas fluorescens***	Swift Current, SK; 12 yr zero-till wheat–canola–pulse system	[90]
**Residue retention**	Delayed emergence	↓ early-season leaf disease	*Kochia scoparia*, *Chenopodium album*; *Alternaria brassicae*, *Leptosphaeria maculans*	Saprophytic fungi; **Streptomyces**; antifungal phenolics; N immobilization	Brandon, MB; 6 yr high-residue wheat–canola rotation	[91]
**Cover crops + grazing**	↓ perennial regrowth	↓ clubroot severity	*Cirsium arvense*; *Plasmodiophora brassicae*	Manure-borne microbes; fungal decomposition; induced systemic resistance	Carman, MB; 3 yr crop–livestock integration	[92]
**High-diversity rotations**	-	↓ root disease buildup	*Fusarium avenaceum*, *Pythium* spp.	Diverse root exudates; AMF colonization; microbial niche competition	Lacombe, AB; 8 yr diversified rotation	[93]

Footnotes: Directional symbols: ↓ = statistically significant reduction relative to conventional practice.

**Table 6:** Effects of RA on reduction in synthetic inputs and sustainability outcomes in Western Canada.

Regenerative Practice	Synthetic N Reduction † (kg N·ha^−1^)	Reduction in the Use of Herbicides, Fungicide and Insecticide	Primary Sustainability Benefits	Representative Locations	References
**No-/reduced till + diversified rotation**	Gradual; cumulative (long-term)	Variable; ↓ under IWM 20% reduction in disease cycle	Soil conservation; SOC gain; reduced runoff and fuel/GHG emissions	Lacombe AB; Swift Current and Scott SK	[50,106]
**Cover crops (short overwintering mixes/overwintering)**	0–15	reduce up to 15% foliar disease pressure indirectly	N retention; soil structure; beneficial insect habitat	SK; AB	[107,108]
**Crop rotation (incl. pulses)**	10–60 ‡ (conservative: 10–20)	Variable Reduce pest pressure	Improved NUE; lower N_2_O intensity	Brooks AB	[50]
**Intercropping (e.g., pea-oat, pea-canola)**	5–15	5~30% lower disease spread	Whole-system resilience; yield stability	Lacombe and Lethbridge AB; Melfort SK; Brandon MB	[103,109]
**Rhizobial inoculation (pulses)**	Tens (variable; fixation-driven)	Indirect; minor effect	Substitutes synthetic N (≤30%)	Lethbridge AB; Swift Current and Canora SK	[50]
**Integrated weed management (IWM) and HWSC**	N/A	Indirect, system-specific	Long-term weed seedbank depletion	Lethbridge AB; Swift Current and Canora SK	[110]
**Split and precision N management**	10–30	Variable, reduced volume via targeted spraying	Reduced N losses and N_2_O emissions	Indian Head SK; PEI	[111,112]
**Cultivar selection and economic thresholds (IPM)**	N/A	10–50 (resistant cultivars + thresholds)	Biodiversity protection: beneficial insects and microbes	Prairie-dominant, multi-site Canada	[113,114]

Footnotes: IWM = integrated weed management; HWSC = harvest weed seed control; NUE = nitrogen use efficiency. Notes: † Synthetic N reduction refers to avoided fertilizer inputs relative to conventional management, not total crop N demand. ‡ Residual N credits are highest following pulse phases; conservative values reflect risk-averse recommendations under Prairie conditions. N/A indicates data not available. ↓ indicates a decrease in the measured value compared to the control.

## Data Availability

The original contributions presented in this study are included in the article/Supplementary Materials. Further inquiries can be directed to the corresponding author.

## Supplementary Materials

The following supporting information can be downloaded at: https://www.mdpi.com/article/10.3390/microorganisms14051075/s1, Table S1. Core principles of regenerative agriculture (RA) mapped to microbial mechanisms and ecosystem outcomes. Table S2. Effects of organic amendments on microbial indicators and soil health. Table S3. Organic amendments and their microbiome-mediated impacts on soil health. Table S4. Mechanisms by which Regenerative Practices suppress Weed, Insect, and Pathogen.

## Abbreviations

The following abbreviations are used in this manuscript:

RA	Regenerative Agriculture
SOC	Soil Organic Carbon
NT	No-Till
RT	Reduced Till
CT	Conventional Tillage
AMF	Arbuscular Mycorrhizal Fungi
VWC	Volumetric Water Content
PAW/PAWC	Plant-Available Water/Plant-Available Water Capacity
PMN	Potentially Mineralizable Nitrogen
MBC/N	Microbial Biomass Carbon/Nitrogen
MAP	Mean Annual Precipitation
RAD	Oilseed Radish
NUE	Nitrogen-Use Efficiency
IWM	Integrated Weed Management
HWSC	Harvest Weed Seed Control
