# Supplementary material for: Soil Microbial Dynamics in Regenerative Agriculture Systems: A Data-Driven Synthesis for Soil Health, Pest Suppression, and Yield Sustainability in the Western Canadian Prairies
Source: Microorganisms. 2026 May 9;14(5):1075. doi: 10.3390/microorganisms14051075 (PMC13210192; doi:10.3390/microorganisms14051075)
Supplement: Supplementary file 1 [file microorganisms-14-01075-s001.zip › microorganisms-4201777-supplementary.pdf]

Supplementary Material

Soil Microbial Dynamics in Regenerative Agriculture Systems: A Data-Driven Synthesis for Soil Health, Pest Suppression, and Yield Sustainability in the Western Canadian Prairies

Susmita Das Nishu[1], M. Nazrul Islam [2*]

[1] Department of Environment and Sustainability, University of Saskatchewan, Saskatoon, SK S7N 5A2, Canada, susmita.nishu@usask.ca (SD Nishu)

[2] College of Graduate and Postdoctoral Studies, University of Saskatchewan, Saskatoon, SK, S7N 5A2, Canada, nazrul.islam@usask.ca (MN Islam)

*Correspondence: nazrul.islam@usask.ca

It contains

Table S1, Table S2, Table S3 and Table S4

Supplementary Tables

Table S1 Core principles of regenerative agriculture (RA) mapped to microbial mechanisms and ecosystem outcomes

**Stimulation**	**Response**	**Outcome**				**Reference**
Principles behind RA Practices	Dominant microbial mechanism	Soil health	Sustainability Contribution	Pest suppression	Study location	
**Minimize soil disturbance (reduced/no-till)**	Preserves fungal hyphae; stabilizes microbial networks	↑ SOC, aggregation, infiltration	Reduced erosion; improved water retention	Reduced pathogen niches via stable microbiomes	Argentin a Slovenia USA	(Díaz-Zorita et al., 2002; Mihelič et al., 2024; Schmidt et al., 2018), (May et al., 2020; Mohammadiani, 2024).
**Maintain soil cover (cover crops)**	Substrate supply to decomposers; ↑ enzymes and biomass	↑ SOM, moisture, mineralizatio n	Reduced runoff; climate buffering	Weed and soilborne pathogen suppression	China USA Argentin a	(Gao et al., 2022; Kim et al., 2020; C.-Y. Liu et al., 2022)
**Maintain living roots year-round (Crop residue perennials, cover crops)**	Continuous rhizodeposition ; microbial phytohormones	↑ SOC, infiltration, water storage	Long-term fertility; ↓ GHG emissions	Competitive exclusion of pathogens	China Canada Australi a	(Gao et al., 2022; Olivia Otchere, 2022) (Aiyer et al., 2022).
**Diversify plant species (crop rotations, polycultures)**	Functional niche expansion; guild diversification	↑ N and P cycling efficiency	Stress resilience (biotic and abiotic)	Disease break; beneficial antagonists	Portugal Canada German y	(Koyama et al., 2022; Rashtbari, 2024; Schmidt et al., 2018; Town et al., 2022)
**Integrate livestock (Holistic**	Dung- and rumen-derived	↑ SOC, nutrient recycling	Closed-loop nutrients; lower inputs	Weed trampling; pest	Canada USA	(Obiora, 2025; Thomas, 2024), (Khatri-Chhetri et

Stimulation	Response	Outcome				Reference
Principles behind RA Practices	Dominant microbial mechanism	Soil health	Sustainability Contribution	Pest suppression	Study location	
**Grazing and Perennial Forage)**	microbial inputs			suppression via food webs		al., 2022), (Rowntree et al., 2020)
**Reduce synthetic inputs**	Preserves microbial consortia; ↑ biomass	Balanced cycling; ↓ residues	Lower pollution; sustained yields	Slower resistance evolution	Canada South Africa Australi a	(Luo et al., 2018) (Khangura et al., 2023) (Dannhauser et al., 2024), (Akanmu et al., 2021), (May et al., 2020; Mohammadiani, 2024).

Note: ↑ increase; ↓ decrease in measured value compared to control.

Table S2 Effects of organic amendments on microbial indicators and soil health

Organic amendment	Microbial biomass	Enzyme activity	Microbial diversity	SOC / SOM	Nutrient cycling	Yield response	Key references
**Manure**	↑↑ (sig.)	↑↑	↑	↑	↑ N, P	↑↑ (sig.)	Luo et al., 2018; Lupwayi et al., 2005
**Compost**	↑ (sig.)	↑	↑	↑↑	↑ retention	↑ (sig.)	Luo et al., 2018; Akanmu et al., 2021
**Crop residues**	↑	↑	↔ / ↑	↑	↓ N (temp.)	↑ (low)	Luo et al., 2018
**Green manure**	↑↑	↑↑	↑	↑	↑ N (fast)	↑	Lin et al., 2025
**Biochar**	↑	↔ / ↑	↑	↑↑ (stable)	↑ retention	↔ / ↑	Akanmu et al., 2021
**Org. vs mineral**	↑↑ (51%)	↑	↑	↑↑	↑ efficiency	↑ (27%)	Luo et al., 2018

Abbreviations: SOC = soil organic carbon; SOM = soil organic matter; ↑ increase; ↓ decrease; ↔ no consistent change; sig. = statistically significant ($p \leq 0.05$). **Note:** Percent values indicate meta-analysis means.

Table S3 Organic amendments and their microbiome-mediated impacts on soil health

**Organic amendments**	**Primary substrate / nutrient features**	**Key microbial dynamics and mechanisms**	**Soil health outcomes linked to microbial emergence**	**System / yield outcomes**	**Evidence** and **quantitative signals**	**Key references**
**Farmyard manure / livestock manure**	Labile C + macro/micronutrients; adds N, P, K; stimulates heterotrophs	Rapid stimulation of microbial biomass and respiration; enhanced enzyme activities; promotes decomposition and nutrient mineralization; pathogen suppression via competitive exclusion and antagonists	Increased microbial biomass C and N; improved nutrient cycling; improved aggregation through microbial byproducts (EPS) and fungal growth	Yield benefits often strong in first years; reduced reliance on mineral fertilizers	Yield +27% vs mineral-only overall; manure strongest yield response (+49%); microbial biomass: MBC +51%, MBN +24% vs mineral-only (meta synthesis); manure increased MBC (cattle up to 3×, +26% to 3×; hog +31%); mineral fertilizer reduced MBC 20–64%; grain yield +75% (hog) and +49% (cattle) in year 1	(Luo et al., 2018; Akanmu et al., 2021; N. Lupwayi et al., 2005; Dannhauser et al., 2024)
**Compost**	Heterogeneous, intermediate decomposition; supports longer SOC accrual	Broadens microbial niche space; increases diversity and functional redundancy; supports sustained enzyme activity and microbial succession	Builds longer-term SOC pools; improved nutrient retention and soil structure via microbial processing	Stable productivity gains; supports long-term resilience	Yield response +17% (compost vs mineral-only); contributes to SOC formation and biological fertility	(Luo et al., 2018; Akanmu et al., 2021)

Organic amendments	Primary substrate / nutrient features	Key microbial dynamics and mechanisms	Soil health outcomes linked to microbial emergence	System / yield outcomes	Evidence and quantitative signals	Key references
**Crop residues (straw/stover)**	High C:N; slow decomposition; can immobilize N temporarily	Stimulates lignocellulose decomposers (fungi, Actinobacteria); increases C-hydrolyzing enzymes; may shift communities toward C acquisition strategies	Improves aggregation and SOC stabilization over time; potential short-term N immobilization affects microbial strategy and crop N availability	Yield response smaller but positive when N is sufficient	Yield response +8% (straw vs mineral-only); enzyme stimulation tied to C hydrolysis; immobilization risk under low N	(Luo et al., 2018; Akanmu et al., 2021; Daunoras et al., 2024)
**Green manures**	Low C:N, labile biomass; rapid mineralization	Rapid microbial growth bursts; stimulates N cycling pathways and enzymes linked to N and P turnover; boosts microbial-driven nutrient release	Short-term increases in microbial biomass and enzyme activity; improved N availability and microbial turnover	Improves subsequent crop N supply; supports rapid fertility restoration	Emphasis on fast microbial response and short-term biomass increases (mechanistic synthesis)	(Lin et al., 2025; Akanmu et al., 2021)
**Biochar**	Recalcitrant C; porous microhabitats; low nutrients	Provides microbial refugia; improves colonization surfaces; increases nutrient retention	Improves moisture retention, C persistence, and microbial	Supports resilience under drought/low SOC	Mechanistic evidence emphasizes niche habitat and retention; market adoption trend	(Akanmu et al., 2021)

**Organic amendments**	**Primary substrate / nutrient features**	**Key microbial dynamics and mechanisms**	**Soil health outcomes linked to microbial emergence**	**System / yield outcomes**	**Evidence and quantitative signals**	**Key references**
	unless co-applied	and microbial habitat stability; effects often strongest with manure/compost co-application	habitat structure; potential increases in microbial diversity/network stability	contexts; yield depends on co-inputs and soil constraints	noted for Canada (contextual)	
**Organic amendments as enzyme regulators (cross-domain)**	Alters pH, SOC, and nutrient availability	Enzyme activity increases across C hydrolysis, N and P decomposition and oxidation; pH and SOC regulate enzyme synthesis and activity	Higher biological functioning; enhanced decomposition and nutrient turnover	Efficient yield response when pH is weak-acidic to weak-alkaline (supports symbiosis)	Enzyme regulation linked to SOC and pH; yield efficiency maximized in weak-acidic to weak-alkaline range	(Daunoras et al., 2024; Agegnehu et al., 2016; Luo et al., 2018)
**Prairie semi-arid constraint context (cross-domain)**	Low SOC and moisture; high climate stress	Organic inputs shift microbial strategies toward improved C/N use efficiency; support resilience and recovery after disturbance	Restores biological integrity; supports stable cycling under drought variability	Promotes long-term sustainability; supports regenerative trajectories	Prairie adoption: manure widely used; survey trend notes increased use	(Dannhauser et al., 2024)

Table S4 Mechanisms by which regenerative practices suppress weed, insect, and pathogen

Practice	Weeds suppression	Insect suppression	Pathogenic suppression	Country	References
**No-tillage / Reduced-tillage (NT/RT)**	Reduced soil disturbance may allow perennial weeds to persist, but surface residue suppresses annual weed germination; long-term suppression is possible with rotation integration.	Stability of soil habitat benefits predator insects and soil arthropods; residue retention provides overwinter habitat for some pest species.	Increase abundance of some pathogen-related microbial genes (nirK, nirS), increase of potential N_2O-producing microbes; disease pressure depends on rotation and residue management.	China, Slovenia, Argentina , Canada, India	Yüze Li et al., 2020; Mihelič et al., 2024; Díaz-Zorita et al., 2002; Yuan et al., 2021; May et al., 2020; Hangs and Schoenau, 2019; Liang et al., 2020; McConkey et al., 2002, 2003; Shi et al., 2024; Lupwayi et al., 2001, 2012; Mangalassery et al., 2015; Mapfumo et al., 2023; Hassani et al., 2024; Kaurin et al., 2018; J. Liu and Lobb, 2021; Statistics Canada, 2021; Helgason et al., 2009b.
**Crop Rotation**	Decrease Weed biomass and pressure; effect enhanced when combined with competitive crops; reduces herbicide reliance.	Diverse rotations disrupt pest life cycles; 3-year canola rotation decrease root maggot, blackleg severity (53–54%); increase predator habitats; reduced pest buildup	Decrease host-specific pathogens (Fusarium, Rhizoctonia solani, Plasmodiophora brassicae) via host break; increase microbial antagonists (Trichoderma, Bacillus, Pseudomonas); crop sequence modifies pathogen vs. beneficial balance.	Canada, Japan, USA, Australia, Bangladesh, India, UK, Shi lanka	Town et al., 2022, 2023; Rashtbari, 2024; O. Otchere, 2022; Koyama et al., 2022; Chen and Liu, 2024; Yang et al., 2024; Yu et al., 2021; Schmidt et al., 2018; Hao et al., 2021; Hedayetullah et al., 2025; Gahagan et al., 2023; M.N. Islam et al., 2024; Peralta et al., 2018; Drury, 2021; J. Zhang et al., 2022; Dukare et al., 2021; Harker et al., 2015; Reed, 2024; Q. Liu et al., 2023; Gallage et al., 2023.

Practice	Weeds suppression	Insect suppression	Pathogenic suppression	Country	References
**Cover Crops**	Decrease weed germination and biomass via shading, residue mulch, allelopathy (e.g., rye, mustard); reduces reliance on herbicides; most effective when terminated at optimal stage to prevent volunteer issues.	Increase habitat for beneficial insects; nectar/pollen resources for natural enemies; potential to disrupt pest cycles; seasonal timing affects efficacy can harbor pests if poorly managed.	Certain Brassicaceae spp. suppress soilborne pathogens via biofumigation. These species (e.g., oilseed radish, alfalfa, phacelia) increase beneficial fungi and may increase some fungal pathotrophs; Sorghum-sudan grass decreases certain pathogens; disease suppression via competition, induced resistance, and soil health improvement.	China, South Korea, Taiwan, Canada, Germany, India, USA, Australia	Gao et al., 2022; Kim et al., 2020; C.-Y. Liu et al., 2022; O. Otchere, 2022; Seitz et al., 2024; Aiyer et al., 2022; Kennedy, 2023; Bowles et al., 2020.
**Organic Amendments (manure, compost, crop residues, green manure, biochar)**	Mixed: green manure and fast-decomposing amendments suppress weeds via shading, residue mulching, and allelopathy; high C:N residues can delay suppression due to slow decomposition; biochar has minimal	Increase habitat and alternative food sources for beneficial arthropods; organic matter incorporation supports detritivore-based food webs; reduced reliance on synthetic inputs may lower secondary pest outbreaks; residue retention may occasionally harbor	Compost and certain manures suppress soilborne pathogens via competition, antibiosis, and improved soil health; biochar may indirectly reduce pathogen incidence via habitat improvement; pathogen suppression strongest with diverse organic inputs; high	China, Canada, Ethiopia	Luo et al., 2018; Akanmu et al., 2021; Dannhauser et al., 2024; Lin et al., 2025; Statistics Canada, 2021; Agegnehu et al., 2016; Daunoras et al., 2024.

Practice	Weeds suppression	Insect suppression	Pathogenic suppression	Country	References
	direct weed control effect.	pest stages if unmanaged.	C:N crop residues may temporarily favor saprophytic fungi.		
**Integrated Crop–Livestock (ICL) (including AMP grazing, bale/swath grazing, perennial forage, multispecies grazing)**	Increase weed suppression via competitive perennial forage cover and trampling; rotational grazing can target weed patches; nutrient-rich manure may stimulate opportunistic weeds if grazing uneven or manure clumps left unmanaged.	Increase Habitat and floral resources for beneficial arthropods; residue and forage cover support detritivores and predators; multispecies grazing may reduce pest buildup via disturbance and vegetation diversity; potential harboring of pests if forage residue unmanaged.	Manure and perennial cover suppress some soilborne pathogens via competition, habitat diversity, and improved soil health; pathogen suppression less documented than in compost-based systems; risk of nutrient leaching, eutrophication, and cyanobacteria if mismanaged.	Australia, Canada, USA, India, UK, Thailand	Sekaran et al., 2021; Bansal et al., 2022; Ominski et al., 2021; Obiora, 2025; Thomas, 2024; Bork et al., 2021; Khatri-Chhetri et al., 2022; Lupwayi et al., 2005; Acosta-Martínez et al., 2010; Rowntree et al., 2020; Gyamfi, 2024; D'Amours et al., 2021; Canadian Cattlemen, 2023; Ijaz et al., 2025.
**Intercropping / Polyculture**	Competitive canopy closure and complementary rooting patterns suppress weeds; less herbicide reliance when well-managed; effectiveness depends on crop pairing and planting density.	Increase floral and habitat diversity supports beneficial insects and predators; crop heterogeneity disrupts pest cycles; may lower specialist pest pressure; effect magnitude depends on crop combinations.	Potential suppression of soilborne pathogens via enhanced microbial diversity, niche competition, and improved soil structure; no strong evidence of pathogen buildup in Prairie trials.	China, Canada	Li et al., 2023; McAuley et al., 2025., Mbanyele, Enesi, Shaw, and Gorim, 2024

Practice	Weeds suppression	Insect suppression	Pathogenic suppression	Country	References
**Commercial Microbial Inoculants (biofertilizers, biocontrol)**	No direct weed suppression; may indirectly reduce weed competitiveness via improved crop vigor and canopy closure.	Indirect effects; healthier plants may better tolerate pest pressure; some microbial biocontrol agents reduce insect-vectored diseases; field evidence in Prairies limited.	Certain inoculants act as biocontrol agents against soilborne pathogens via competition, induced systemic resistance, and root colonization; effectiveness varies by strain, crop, and soil conditions.	Canada, China, India, USA	Laskar et al., 2024; Hynes et al., 1995.Timofeeva, Galyamova, and Sedykh, 2024, Biswaray, 2015
